# Synergistic potential of antibiotics against an *in vitro* multispecies biofilm model for peri-implantitis

**DOI:** 10.3389/fbioe.2026.1800253

**Published:** 2026-04-13

**Authors:** Neele Brümmer, Karolin Behrens, Katharina Doll-Nikutta, Philipp-Cornelius Pott, Meike Stiesch

**Affiliations:** 1 Clinic of Prosthetic Dentistry and Biomedical Materials Research, Hannover Medical School, Hannover, Germany; 2 Lower Saxony Center for Biomedical Engineering, Implant Research and Development (NIFE), Hannover, Germany

**Keywords:** amoxicillin, antibiotic, doxycycline, *in vitro*, metronidazole, minocycline, multispecies biofilm, peri-implantitis

## Abstract

**Background:**

Despite their widespread success, dental implants remain vulnerable to biofilm-associated infections such as peri-implantitis. Local antibiotic (AB) application may enhance treatment outcomes; however, its use remains controversial due to limited evidence and the lack of standardized recommendations regarding active agents and effective concentrations. This study aimed to identify potent antibiotic combinations and concentrations against a peri-implantitis-associated multispecies biofilm (MB) *in vitro* and to assess the influence of implant material on their efficacy.

**Method:**

An oral multispecies biofilm model (MSBM) was cultivated in the presence of the antibiotics amoxicillin (Amox), doxycycline (Doxy), minocycline (Mino), and metronidazole (Metro), both as single agents and in combination with Metro at varying concentrations. Synergistic effects were assessed by turbidity measurement, Bactiter Glo™ assay and Resazurin assay. The most effective concentrations were further examined using confocal laser scanning microscopy. Additionally, they were tested on three potential implant materials: titanium grade 4, titanium grade 5, and an experimental ultrafine-grained niobium alloy and on a mature biofilm.

**Results:**

Amox, Doxy and Mino demonstrated strong efficacy against the MB, whereas Metro alone showed little to no effect. Synergistic interactions were mainly observed when comparing to Metro’s limited activity. A tendency toward enhanced efficacy of Amox and Doxy in combination with Metro was noted, although not statistically significant. The antibacterial performance of all agents was independent of the implant material and reduced when applied on mature biofilm.

**Conclusion:**

These findings highlight the potential of locally applied Amox and Doxy, alone or in combination with Metro, as a targeted approach for peri-implantitis management and indicate that their effectiveness is largely independent of implant materials. Further studies using *in vivo* biofilms are warranted to optimize antibiotic combinations and concentrations for clinical application.

## 1 Introduction

In modern dentistry, dental implants have become a standard and well-established method for replacing missing teeth, demonstrating high success rates as a result of extensive research. Despite their numerous advantages and proven efficacy, dental implants remain susceptible to complications, predominantly due to bacterial biofilms (Derks and Tomasi, 2015). Planktonic bacteria are ubiquitously present in the oral cavity. When coming in contact with solid surfaces, biofilms generally form on the interface to the moist environment. The accumulation of pathogenic bacteria on implant surfaces can initially lead to an inflammation of the periimplant soft tissues, known as mucositis. If the biofilm persists, depending on the host response, mucositis may progress to peri-implantitis, a condition characterized by the gradual loss of the implant-supporting bone. Ultimately, peri-implantitis can lead to implant loss. With a prevalence of 22% after 10 years, peri-implantitis already emerged as one of the major challenges in contemporary dentistry (Derks and Tomasi, 2015; Dreyer et al., 2018). Due to the increasing number of implant placements and in the context of an aging population, the prevalence and clinical significance of this condition are expected to become even more relevant in the future.

Although various implant- and host-related risk factors exist for peri-implantitis, pathogenic biofilm remains the trigger for the actual inflammation (Dreyer et al., 2018; Fu and Wang, 2020). Biofilms are composed of microorganisms embedded in an extracellular matrix consisting of polysaccharides, proteins, glycolipids, and bacterial DNA, most of which they produce themselves. Within biofilms, microorganisms benefit from enhanced resistance to various environmental stresses, including nutrient deficiency, mechanical shear forces, pH fluctuations, and antimicrobial agents. Healthy peri-implant sites are predominantly colonized by aerobic and facultative anaerobic bacteria such as *Streptococcus*, *Rothia*, *Neisseria* and *Corynebacterium* species (Joshi et al., 2025b). Furthermore, *Veilonellae* and *Actinomyces* species belong to the early colonizers of implant surfaces (Colombo and Tanner, 2019). The severity of peri-implant inflammation increases as biofilm diversity and the number of pathogenic and anaerobic bacteria increase (Al-Ahmad et al., 2018; Joshi et al., 2025a; Joshi et al., 2025b). According to a recent review by *Carvalho* et al., peri-implantitis is associated with the presence of *Porphyromonas gingivalis* (*P. gingivalis*), *Tannerella forsythia* (*T. forsythia*), *Treponema denticola* (*T. denticola*), *Fusobacterium nucleatum* (*F. nucleatum*), *Prevotella intermedia* (*P. intermedia*) and *Staphylococcus epidermidis* (*S. epidermidis*) (Carvalho É et al., 2023). Organized in a biofilm, these microorganisms can trigger inflammatory processes at the implant site through various virulence factors.

In addition to biological factors, the implant material itself significantly influences the development of peri-implantitis. Variations in material composition and surface structures can modulate bacterial accumulation at the implant site (Yu et al., 2024). Nowadays, dental implants are primarily made of grade 4 and 5 titanium (Hanawa, 2020). For patients with titanium allergies, implants made of zirconia are available; however, they exhibit reduced mechanical strength compared to titanium (Sivaraman et al., 2018). An experimental ultrafine-grained niobium alloy could be a promising alternative, offering high mechanical strength combined with a markedly low allergenic potential (Rubitschek et al., 2012; Brümmer et al., 2024; Pott et al., 2025). While the influence of implant material composition on the development of peri-implantitis is well known, its impact on the outcomes of peri-implantitis therapy remains insufficiently explored. Considering the expanding range of implant materials available, this aspect deserves increased focus in research.

The primary objective of prevention of peri-implantitis is to halt the formation of biofilms on implant surfaces before the pathogenic shift occurs. In case of diagnosed peri-implantitis, treatment options include both non-surgical and surgical procedures, with the main goal of biofilm removal. Additionally, adjuvant therapies, including locally and systematically applied antibiotics, topical antimicrobials, photodynamic or laser therapy are being used to further reduce the bacterial load (Schwarz et al., 2022; Liñares et al., 2023; Giok et al., 2024). Literature reviews show widely varying, yet generally unsatisfactory, success rates for peri-implantitis treatments, ranging from 33% to 75% (Schwarz et al., 2022; Acar and Guncu, 2024). At the same time, current literature reviews emphasize the potential benefits of adjuvant antibiotic therapy enhancing the outcomes of both non-surgical and surgical debridement (Wang et al., 2022a; Okuma-Oliveira et al., 2024). However, despite those promising findings, international guidelines currently advise against the routine use of antibiotics in peri-implantitis management due to increasing concerns regarding antibiotic resistance development (Herrera et al., 2023). Consequently, further research is imperative to optimize antibiotic regimes, including the development of appropriate dosage forms and concentrations, in order to improve therapeutic outcomes in peri-implantitis treatment.

The combination of amoxicillin (Amox) and metronidazole (Metro), when administered systematically, currently holds the highest evidence base among antibiotic therapies for peri-implantitis (Schwarz et al., 2022; Liñares et al., 2023; Giok et al., 2024). Despite the benefits in treatment outcomes, patients treated with systemic antibiotics can suffer from undesirable side effects on the gastrointestinal microflora (Maier et al., 2018). Locally applied antibiotics exhibit fewer side effects and show higher local drug concentrations but can show a reduced efficacy due to clearance by saliva and sulcular fluid from the peri-implant pocket on the one side and poor penetration into deep mucosa on the other side (Nastri et al., 2019; Nakajima et al., 2021). Nevertheless, the advantages of topical therapies outweigh if an appropriate and effective agent is used. Various agents have been employed for this purpose in the literature, including doxycycline (Doxy) (Büchter et al., 2004), minocycline (Mino) (Salvi et al., 2007; Renvert et al., 2008; Bassetti et al., 2014; Cha et al., 2019), and Metro either alone (Al-Khureif et al., 2020) or in combination with Amox (Ahmed et al., 2020). However, based on the existing evidence, no definitive conclusion can be drawn regarding the optimal antibiotic agent, combination or concentration for adjuvant local treatment of peri-implantitis.

The aim of this study was therefore to evaluate the efficacy of various antibiotics, applied in different combinations and at different concentrations, on different developmental stages of an oral multispecies peri-implant biofilm model *in vitro*. In addition, the study investigated how different implant materials influence the effectiveness of these antibiotics.

## 2 Materials and methods

**FIGURE 1:**
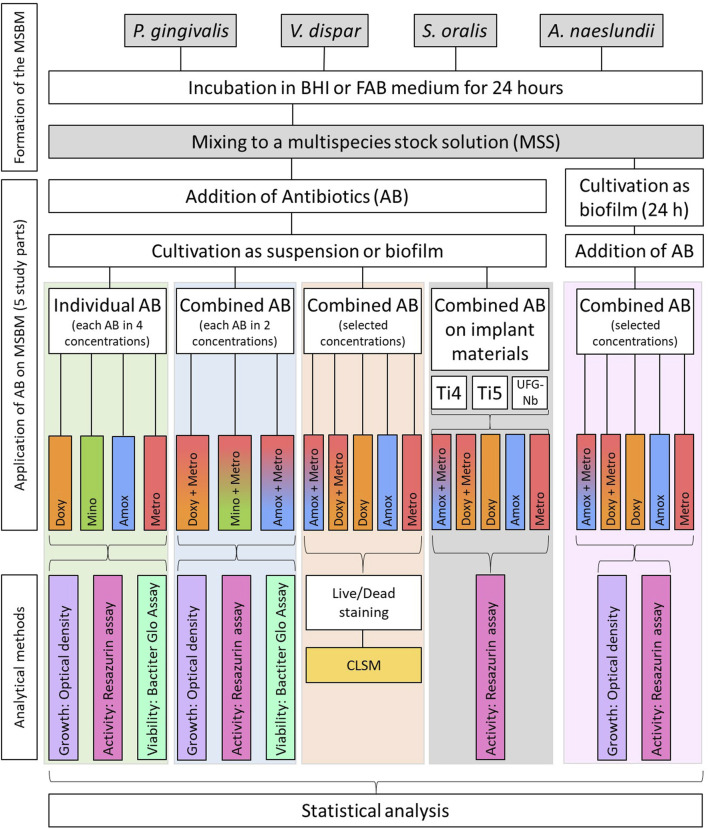
Flowchart of the experimental procedure. The five study parts are highlighted in different colors. Doxy = doxycycline, Mino = minocycline, Amox = amoxicillin, Metro = metronidazole, CLSM = Confocal Laser Scanning Microscopy.

This *in vitro* study was conducted on different developmental stages (planktonic suspension and mature biofilm) of an established oral multispecies biofilm model (MSBM) consisting of the bacteria *Porphyromonas gingivalis* (*P. gingivalis*), *Veilonella dispar* (*V. dispar*), *Streptococcus oralis* (*S. oralis*) and *Actinomyces naeslundii* (*A. naeslundii*). First, the efficacy of the four antibiotics Doxy, Mino, Amox and Metro, administered in different concentrations, were tested on the MSBM using standard turbidity measurement and viability assays. Afterwards, Doxy, Mino and Amox were each combined with Metro and the effects on the MSBM were analyzed. The effects of the most promising combinations were further analyzed using confocal laser scanning microscopy (CLSM). Additionally, they were also applied on the multispecies biofilm in presence of three potential implant materials: titanium grade 4 (Ti4), titanium grade 5 (Ti5) and an experimental ultrafine-grained niobium alloy (UFG-Nb) and on mature biofilm, that has been cultivated in advance. Each experiment was conducted in triplicate with three biological replicates (n = 9). For the part of the study involving implant materials, two biological replicates were conducted (n = 6). Figure 1 provides an overview over the experimental procedure.

### 2.1 Formation of the multispecies biofilm model (MSBM)

The multispecies biofilm model (MSBM) was formed as described by Kommerein et al. (2017). Briefly, *S. oralis* ATCC® 9811, *A. naeslundii* DSM 43013, *V. dispar* DSM 20735 and *P. gingivalis* DSM 20709 were acquired from the German Collection of Microorganisms and Cell Cultures (DSMZ) and the American Type Culture Collection (ATCC) and incubated for 24 h at 37 °C under anaerobic conditions. Incubation of *P. gingivalis* took place in Fastidious Anaerobe Broth (FAB, Neogen, Lansing, Michigan, United States), the other bacterial strains were incubated in Brain Heart Infusion (BHI, Oxoid, Wesel, Germany) supplemented with 10 μg/mL Vitamik K (VitK). After incubation, equal volumes of each species were mixed with BHI + VitK to achieve an optical density (600 nm) of 0.1. 2 mL of each species solution were mixed in order to form the multispecies stock solution (MSS). The MSS was added to 96-well plates and mixed with the respective AB. The plates were incubated for 24 h at 37 °C under anaerobic conditions standing to allow for multispecies biofilm (MB) formation or on a shaker at 500 rpm, in order to create a multispecies suspension (MS).

### 2.2 Antibiotics tested individually on bacterial growth and viability

Each of the selected antibiotics amoxicillin (Sigma-Aldrich Amoxicillin A8523-1G, Darmstadt, Germany), minocycline (Sigma-Aldrich Minocyclin-hydrochlorid M9511, Darmstadt, Germany), doxycycline (Sigma-Aldrich Doxycyclin hyclate D9891-1G, Darmstadt, Germany) and metronidazole (Sigma-Aldrich Metronidazole M1547 BioXtra, Darmstadt, Germany) was added to the MSS in four different concentrations according to Table 1. Chlorhexidine (CHX) was added to the bacteria as negative control.

**TABLE 1:** Composition of the experiments for antibiotics tested individually on MS and MB.

No	Experimental group AB concentration in µg/mL	Antibiotic amount and concentration	MSS in µl	BHI + VitK in µl
1	Amox 0.25	10 μL Amox 25 μg/mL	100	890
2	Amox 0.04	10 μL Amox 4 μg/mL	100	890
3	Amox 0.004	10 μL Amox 0.4 μg/mL	100	890
4	Amox 0.0004	10 μL Amox 0.04 μg/mL	100	890
5	Metro 1000	50 μL Metro 20 mg/mL	100	850
6	Metro 600	50 μL Metro 12 mg/mL	100	850
7	Metro 300	50 μL Metro 6 mg/mL	100	850
8	Metro 100	50 μL Metro 2 mg/mL	100	850
9	Mino 32	50 μL Mino 0.64 mg/mL	100	850
10	Mino 0.25	50 μL Mino 5 μg/mL	100	850
11	Mino 0.025	50 μL Mino 0.5 μg/mL	100	850
12	Mino 0.0025	50 μL Mino 0.05 μg/mL	100	850
13	Doxy 4	10 μL Doxy 400 μg/mL	100	890
14	Doxy 1	10 μL Doxy 100 μg/mL	100	890
15	Doxy 0.25	10 μL Doxy 25 μg/mL	100	890
16	Doxy 0.05	10 μL Doxy 5 μg/mL	100	890
17	CHX 0,001%	10 μL CHX 0,12%	100	890
18	Growth control	-	100	900
19	Medium control	-	-	1000

*No, number; MSS, multispecies stock solution; Amox, amoxicillin; Metro, metronidazole; Mino, minocycline; Doxy, doxycycline; CHX, chlorhexidine.*

The procedure was performed identically for MS and MB experiments. Afterwards, optical density of both plates was measured at a plate reader (TECAN Infinite M200 Pro, Männedorf, Switzerland) to assess bacterial growth. Bacterial vitality was evaluated for the MS plate using the BacTiter-Glo™ Assay (Promega BacTiter-Glo™ Microbial Cell Viability Assay, Madison, Wisconsin, United States). Briefly, 50 μL MS were transferred to an opaque well plate to ensure light protection. The BacTiter-Glo™ reagent was prepared as instructed by the manufacturer and 50 µL were added to each well. Incubation took place in a dark place at room temperature for 5 min before luminescence was measured. For bacterial activity analysis, a resazurin assay was applied on the MB plate. Therefore, every well was washed twice with BHI + VitK before 100 µL of a 0,01% resazurin suspension (1 mL 0,1% Resazurin (Resazurin, Sigma Aldrich, Merck KGaA, Darmstadt, Germany) + 9 mL BHI + VitK) was added per well. After 30 min of anaerobic incubation at 37 °C, fluorescence was measured with the plate reader.

### 2.3 Antibiotics tested in combinations on bacterial growth and viability

The lowest concentration of each antibiotic tested in the first study part that still allowed (reduced) bacterial growth and vitality was defined as the partial inhibitory concentration (IC_P_) and the concentration below that one (with full bacterial growth and viability) was defined as the subinhibitory concentration (IC_S_). In order to reveal synergistic effects between the antibiotics, IC_P_ and IC_S_ of Amox, Mino and Doxy were combined each with IC_P_ and IC_S_ of Metro. Table 2 provides an overview over the experimental conditions.

**TABLE 2:** Composition of the experiments for antibiotics tested in combinations on MS and MB.

No	Experimental group Concentration AB 1/AB 2 in µg/mL	Metro amount and concentration	2nd AB amount and concentration	MSS in µl	BHI + VitK in µl
1	Metro IC_P_/Amox IC_P_ Metro 600/Amox 0.04	50 µL Metro 12 mg/mL	10 μL Amox 4 μg/mL	100	840
2	Metro IC_S_/Amox IC_P_ Metro 300/Amox 0.04	50 µL Metro 6 mg/mL	10 μL Amox 4 μg/mL	100	840
3	Metro IC_P_/Amox IC_S_ Metro 600/Amox 0.004	50 µL Metro 12 mg/mL	10 μL Amox 0.4 μg/mL	100	840
4	Metro IC_S_/Amox IC_S_ Metro 300/Amox 0.004	50 µL Metro 6 mg/mL	10 μL Amox 0.4 μg/mL	100	840
5	Metro IC_P_/Mino IC_P_ Metro 600/Mino 0.025	50 µL Metro 12 mg/mL	50 µL Mino 0.5 μg/mL	100	800
6	Metro IC_S_/Mino IC_P_ Metro 300/Mino 0.025	50 µL Metro 6 mg/mL	50 µL Mino 0.5 μg/mL	100	800
7	Metro IC_P_/Mino IC_S_ Metro 600/Mino 0.0025	50 µL Metro 12 mg/mL	50 µL Mino 0.05 μg/mL	100	800
8	Metro IC_S_/Mino IC_S_ Metro 300/Mino 0.0025	50 µL Metro 6 mg/mL	50 µL Mino 0.05 μg/mL	100	800
9	Metro IC_P_/Doxy IC_P_ Metro 600/Doxy 0.25	50 µL Metro 12 mg/mL	10 µL Doxy 25 μg/mL	100	840
10	Metro IC_S_/Doxy IC_P_ Metro 300/Doxy 0.25	50 µL Metro 6 mg/mL	10 µL Doxy 25 μg/mL	100	840
11	Metro IC_P_/Doxy IC_S_ Metro 600/Doxy 0.05	50 µL Metro 12 mg/mL	10 µL Doxy 5 μg/mL	100	840
12	Metro IC_S_/Doxy IC_S_ Metro 300/Doxy 0.05	50 µL Metro 6 mg/mL	10 µL Doxy 5 μg/mL	100	840
13	CHX 0,001%	10 μL 0,12% CHX	-	100	890
14	Growth control	-	-	100	900
15	Medium control	-	-	-	1000

*No, number; MSS, multispecies stock solution; Amox, amoxicillin; Metro, metronidazole; Mino, minocycline; Doxy, doxycycline; CHX, chlorhexidine; IC_P_, partial inhibitory concentration; IC_S_, subinhibitory concentration.*

The antibiotic combinations were mixed with the MSS and incubated under the same conditions as the individual antibiotics. Subsequently, optical density was measured, followed by analysis using the BacTiter-Glo™ assay and the resazurin assay as described previously.

### 2.4 CLSM analysis of biofilms

The most effective AB combinations identified in the previous experiments were further analyzed using confocal laser scanning microscopy (CLSM) following LIVE/DEAD staining. For this purpose, MB was prepared as previously described and treated with individual AB and combined AB, as outlined in Table 3.

**TABLE 3:** Composition of the experiments for CLSM analysis.

No	Experimental group Concentration AB 1/AB 2 in µg/mL	Metro amount and concentration	2nd AB amount and concentration	MSS in ul	BHI + VitK in ml
1	Metro IC_S_/Amox IC_P_ Metro 300/Amox 0.04	250 µL Metro 6 mg/mL	50 μL Amox 4 μg/mL	500	4.2
2	Amox IC_P_ Amox 0.04	-	50 μL Amox 4 μg/mL	500	4.45
3	Metro IC_s_ Metro 300	250 µL Metro 6 mg/mL	-	500	4.25
4	Metro IC_S_/Doxy IC_P_ Metro 300/Doxy 0.25	250 µL Metro 6 mg/mL	50 µL Doxy 25 μg/mL	500	4.2
5	Doxy IC_P_ Doxy 0.25	-	50 µL Doxy 25 μg/mL	500	4.45
6	CHX 0,001%	50 μL CHX 0,12%	-	500	4.45
7	Growth control	-	-	500	4.5
8	Medium control	-	-	-	5

*No, number; MSS, multispecies stock solution; Amox, amoxicillin; Metro, metronidazole; Mino, minocycline; Doxy, doxycycline; CHX, chlorhexidine; IC_P_, partial inhibitory concentration; IC_S_, subinhibitory concentration.*

After anaerobic incubation of the well plates for 24 h at 37 °C the supernatant was removed and the wells were washed twice with BHI + VitK. A LIVE/DEAD staining solution was prepared by adding 12.5 µL of Syto9 and 12.5 µL of Propidiumiodid (both Invitrogen LIVE/DEAD BacLight Bacterial Viability; Waltham, Massachusetts, United States) to 25 mL Phosphate Buffered Saline (PBS; Sigma-Aldrich, Darmstadt, Germany). 3 mL of the staining solution were added to each well. Incubation took place at room temperature for 15 min in a dark place. Separately, a Glutardialdehyde suspension was mixed from PBS and Glutardialdehyde (Carl Roth Glutardialdehyd 25%, Karlsruhe, Germany) in a ratio of 9:1 and 3 mL were added to each well. After incubation for 15 min at 4 °C, die supernatants were removed and 3 mL PBS were added per well. Analysis of the biofilm was performed with a CLSM (Leica DM6 CS microscope, Leica Microsystems GmbH, Wetzlar, Germany) and the Leica Application Suite X (LAS Xsoftware, Leica Microsystems GmbH, Wetzlar, Germany). A lens with 40x magnification was used to create 5 pictures at defined localizations (middle, up, down, left, right) in a 1024 × 1024 pixels resolution. Syto9 was excited with a 488 nm laser and emission was recorded from 500–550 nm. PI was excited simultaneously with a 552 nm laser and emission was recorded from 650–750 nm. Image analysis was done qualitatively by creating overlay images within the same software. Biofilm volume and live/dead distribution was calculated for each image (n = 15) using the Imaris x64 8.4.2 software package (Bitplane AG, Zurich, Switzerland).

### 2.5 Antibiotics tested on implant materials

Finally, the efficacy of two antibiotic combinations and their individual components was evaluated in the presence of three potential dental implant materials. For this purpose, commercially available grade 4 (Ti4) and grade 5 (Ti5) titanium were used, along with an experimental ultrafine-grained niobium alloy (UFG-Nb), developed and provided by the group of Maier et al. at the Institute of Materials Science, Leibniz University Hannover, Germany. The AB were mixed with the MSS as specified in Table 4 and added to wells containing two discs of one of the three implant materials, each with a diameter of 5 mm and a height of 1 mm. After anaerobic MB incubation for 24 h at 37 °C, the resazurin assay was performed as previously described. For visualization, one sample per material was randomly selected and imaged using a scanning electron microscope (SEM) in order to examine the surface properties in more detail. SEM images were generated at two magnifications (300x and 2000x) using a Zeiss EVO MA 10 (Carl Zeiss, Oberkochen, Germany). The following acquisition parameters were used: 300x Images (EHT = 15.00kV, WD 10.0 mm, Width 1 mm), 2000x Images (EHT = 15.00kV, WD 10.0 mm, Width 150 µm), 2000x Images (EHT = 15.00kV, WD 10.0 mm, Width 150 µm).

**TABLE 4:** Composition of the experiments on implant materials.

No	Experimental group Concentration AB 1/AB 2 in µg/mL	Metro amount and concentration	2nd AB amount and concentration	MSS in ul	BHI + VitK in µl	Material
1	Metro IC_S_/Amox IC_P_ Metro 300/Amox 0.04	50 µL Metro 6 mg/mL	10 μL Amox 4 μg/mL	100	840	Ti4/Ti5/UFG-Nb
2	Amox IC_P_ Amox 0.04	-	10 μL Amox 4 μg/mL	100	890	Ti4/Ti5/UFG-Nb
3	Metro IC_s_ Metro 300	50 µL Metro 6 mg/mL	-	100	850	Ti4/Ti5/UFG-Nb
4	Metro IC_S_/Doxy IC_P_ Metro 300/Doxy 0.25	50 µL Metro 6 mg/mL	10 µL Doxy 25 μg/mL	100	840	Ti4/Ti5/UFG-Nb
5	Doxy IC_P_ Doxy 0.25	-	10 µL Doxy 25 μg/mL	100	890	Ti4/Ti5/UFG-Nb
6	CHX 0,001%	10 μL 0,12% CHX	-	100	890	Ti4/Ti5/UFG-Nb
7	Growth control	-	-	100	900	Ti4/Ti5/UFG-Nb
8	Medium control	-	-	-	1000	Ti4/Ti5/UFG-Nb

*No, number; MSS, multispecies stock solution; Amox, amoxicillin; Metro, metronidazole; Mino, minocycline; Doxy, doxycycline; CHX, chlorhexidine; IC_P_, partial inhibitory concentration; IC_S_, subinhibitory concentration; Ti4, titanium grade 4; Ti5, titanium grade 5; UFG-Nb, ultrafine-grained niobium alloy.*

### 2.6 Antibiotics tested on mature biofilm

To better mimic the therapeutic situation, the most promising AB combinations and their components were further applied onto a mature biofilm and analyzed accordingly. Therefore, the MSS was prepared as described previously and mixed with BHI (ratio 1:9). After anaerobic incubation for 24 h at 37 °C, 100 µL of the respective AB-solutions were added to the biofilm. The applied concentrations are displayed in Table 5. Again, anaerobic incubation was performed for 24 h at 37 °C. Afterwards, optical density was measured and bacterial activity was determined using a resazurin assay as previously described.

**TABLE 5:** Composition of the experiments on mature biofilm.

No	Experimental group Concentration AB 1/AB 2 in µg/mL	Metro amount and concentration	2nd AB amount and concentration	BHI + VitK in µl
1	Metro IC_S_/Amox IC_P_ Metro 300/Amox 0.04	50 µL Metro 6 mg/mL	10 μL Amox 4 μg/mL	940
2	Amox IC_P_ Amox 0.04	-	10 μL Amox 4 μg/mL	990
3	Metro IC_s_ Metro 300	50 µL Metro 6 mg/mL	-	950
4	Metro IC_S_/Doxy IC_P_ Metro 300/Doxy 0.25	50 µL Metro 6 mg/mL	10 µL Doxy 25 μg/mL	940
5	Doxy IC_P_ Doxy 0.25	-	10 µL Doxy 25 μg/mL	990
6	CHX 0,001%	10 μL CHX 0,12%	-	990
7	Growth control	-	-	1000
8	Medium control	-	-	1000

*No, number; MSS, multispecies stock solution; Amox, amoxicillin; Metro, metronidazole; Mino, minocycline; Doxy, doxycycline; CHX, chlorhexidine; IC_P_, partial inhibitory concentration; IC_S_, subinhibitory concentration.*

### 2.7 Statistical analysis

The data were normalized to the growth control. The statistical analysis was performed with GraphPad Prism Version 10.3 (GraphPad Prism, GraphPad Software, Boston, Massachusetts, United States). The normal distribution of the data was confirmed using the D’Agostino and Pearson test. The one sample Wilcoxon test was used to investigate efficacy of the single ABs. To assess the synergistic effects of AB combinations and to compare biofilm volume between the experimental groups, the Kruskal–Wallis test was applied. Results on the implant materials were compared using a two-way ANOVA. The level of significance was set at α = 0.05.

## 3 Results

### 3.1 Efficacy of single antibiotics and definition of IC_P_ and IC_S_

**FIGURE 2:**
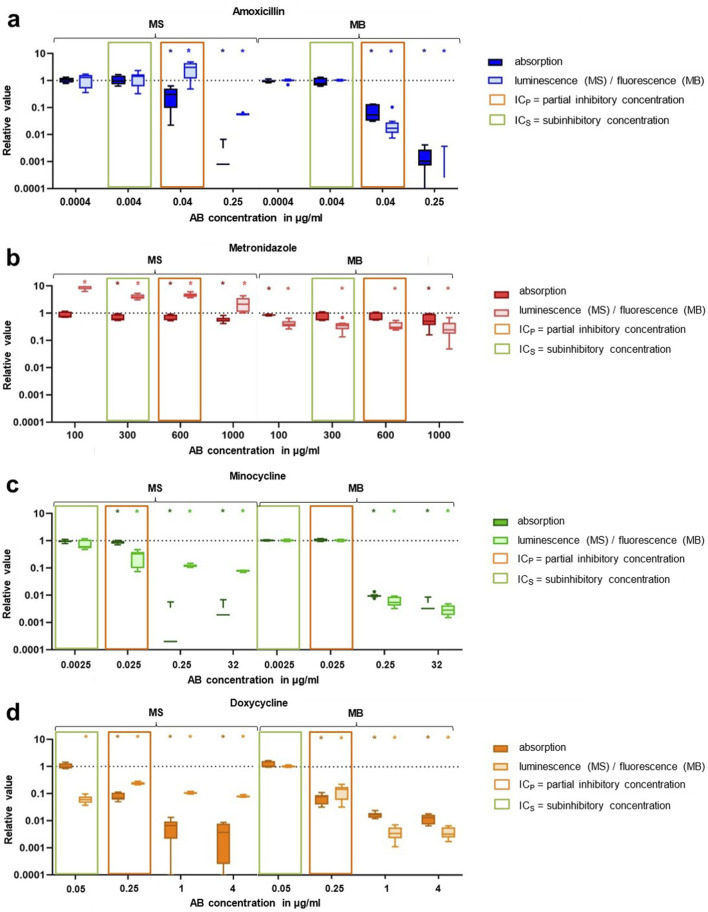
Results of bacterial growth and viability after 24 h treatment with Amox **(a)**, Metro **(b)**, Mino **(c)**, Doxy **(d)** examined individually given as Tukey boxplots. MS = multispecies suspension, MB = multispecies biofilm, AB = antibiotic, * = significant difference (p ≤ 0.05).

The results of the experiments in which the individual antibiotics were examined are shown in Figures 2a–d. Subsequently, the lowest concentration that already showed a certain antibacterial effect on MS and MB was defined as the partial inhibitory concentration (IC_P_) and the concentration below that one was defined as subinhibitory concentration (IC_S_).

In the presence of amoxicillin (Amox), bacterial growth, as measured by absorption, decreased significantly at a concentration of 0.04 μg/mL (p = 0.004), accompanied by a reduction in fluorescence (bacterial activity). At this concentration, luminescence (bacterial viability) increased significantly (p = 0.02). Accordingly, the IC_P_ for Amox was set at 0.04 μg/mL, and the IC_S_ was established at 0.004 μg/mL. The results for metronidazole (Metro) showed a low but still significantly reduced absorption from 300 μg/mL when applied to MS (p = 0.004). Bacterial viability (luminescence) increased significantly at all concentrations, while bacterial activity (fluorescence) decreased with every applied concentration. Compared to the other AB, the differences between the various concentrations remained relatively small. Consequently, IC_P_ was set at 600 μg/mL and IC_S_ was set at 300 μg/mL. Minocycline (Mino), when applied on MS, demonstrated significantly decreased absorption and luminescence values for concentrations of 0.025 μg/mL and higher (p ≤ 0.008). However, with MB, significant reductions in absorption and fluorescence were observed only at a higher concentration of 0.25 μg/mL (p = 0.004). The IC_P_ for Mino was set at 0.025 μg/mL, and the IC_S_ was established at 0.0025 μg/mL. Doxycycline (Doxy) resulted in reduced absorption on both MS and MB starting at a concentration of 0.25 μg/mL (p = 0.04). Fluorescence also decreased from 0.25 μg/mL whereas luminescence already showed significant reductions at a lower concentration of 0.05 μg/mL. Thus, the IC_P_ for Doxy was set at 0.25 μg/mL, and the IC_S_ was established at 0.05 μg/mL.

### 3.2 Combinations with metro show synergistic effects

**FIGURE 3:**
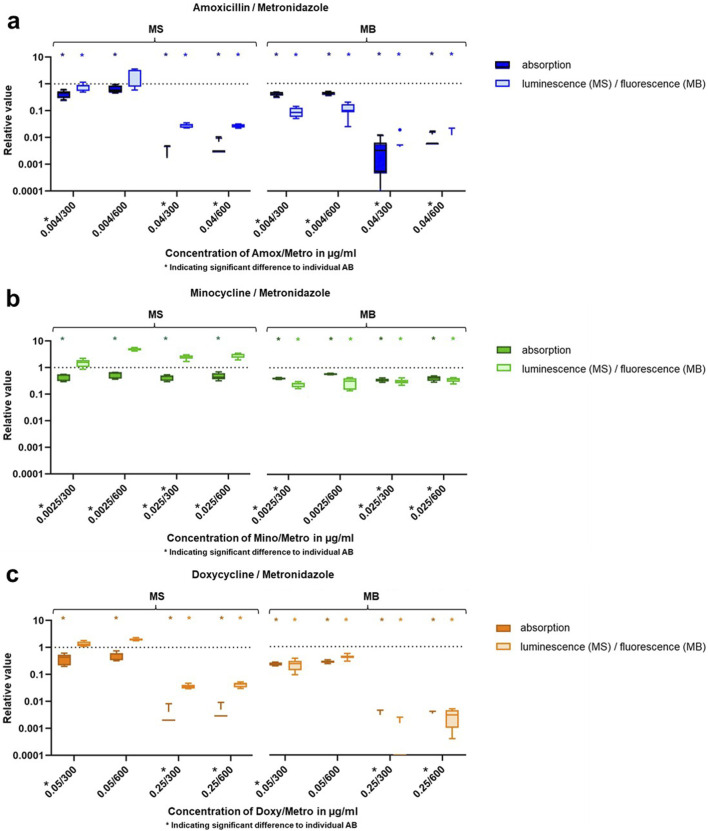
Results of bacterial growth and viability after 24 h treatment with Metro combined with Amox **(a)**, Mino **(b)** and Doxy **(c)** given as Tukey boxplots. MS = multispecies suspension, MB = multispecies biofilm, * = significant difference (p ≤ 0.05).

Results of the experiments with IC_P_ and IC_S_ of Amox, Mino and Doxy each combined with IC_P_ and IC_S_ of Metro are shown in Figure 3.

The combination of Amox IC_S_ with both concentrations of Metro resulted in a slight reduction in absorption. However, only the combinations including Amox IC_P_ caused a reduction beyond a relative value of 0.1. Relevant decreases in luminescence and fluorescence were also observed exclusively in combinations containing Amox IC_P_. The application of Metro’s IC_P_ did not produce any noticeable changes compared to its IC_S_. However, compared to the individual AB, the combinations of 0.004/300 (on MS and MB) and 0.004/600 (on MB) demonstrated superior efficacy relative to Amox alone at the same concentration. The combination of 0.04 μg/mL Amox with Metro resulted in reduced absorption, luminescence and fluorescence compared to Amox alone. However, these reductions were not statistically significant. Nevertheless, all experiments involving 0.04 μg/mL Amox combined with Metro yielded significantly better results than those with Metro alone.

All tested combinations of Mino and Metro resulted in reduced absorption; however, none of the relative values fell below 0.34 (observed at 0.025/300 on MB). Fluorescence was similarly reduced across all combinations, whereas luminescence increased for all. No relevant differences were observed between the various combinations. Compared to Mino tested individually, the combination with Metro yielded improved results at all concentrations, except for 0.0025/600 on MB. Only the combination 0.25/300 (on MS and MB) and 0.25/600 (on MB) achieved better results than the respective concentration of Metro tested individually.

Every applied combination of Doxy and Metro resulted in decreased absorption values. However, only the combinations containing Doxy IC_P_ achieved a reduction in absorption, luminescence, and fluorescence below a relative value of 0.1. Between the IC_P_ and IC_S_ of Metro, no relevant difference could be seen. Compared to the equally concentrated Doxy tested individually, both combinations with Doxy IC_P_ demonstrated better efficacy against MS and MB; however, these findings were not statistically significant. Only the combination 0.05/300 showed significant superior efficacy then Doxy alone. Yet, both combinations with Doxy IC_P_ demonstrated significant better results than equally concentrated Metro alone.

### 3.3 Biofilm analysis by CLSM

**FIGURE 4:**
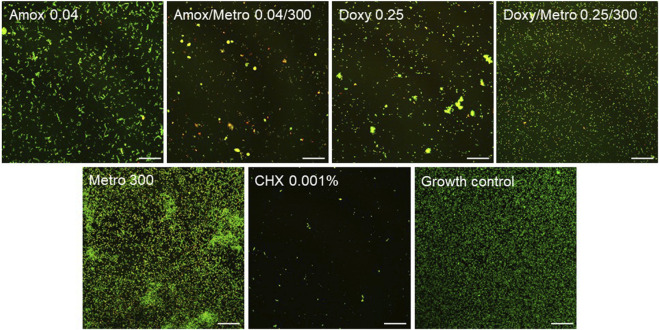
Representative CLSM visualization following LIVE/DEAD staining of biofilm treated with different AB specified in µg/mL. CHX 0.001% = Chlorhexidine 0.001% used as negative control. Living cells are stained in green, whereas dead cells are stained in red. Scale bars represent 50 µm.

The most effective combinations identified in the previous experiments–Amox/Metro 0.04/300 and Doxy/Metro 0.25/300 – as well as their respective single-antibiotic components, were subjected to additional qualitative CLSM analysis following LIVE/DEAD staining. Figure 4 shows representative images taken at 40x magnification.

Living cells are stained in green, whereas dead cells are stained in red. The growth control exhibits a homogeneously distributed biofilm without evidence of cluster formation and is predominantly composed of living cells. The microbial morphology is mainly characterized by small cocci (presumably *S. oralis*), interspersed with individual rod-shaped bacteria, that could be *A. naeslundii* and large cocci (probably *V. dispar*). In contrast, images obtained after treatment with Amox and Doxy, either alone or in combination with Metro, demonstrate reduced overall cell numbers and an increased proportion of dead cells. The Amox-treated biofilm shows a homogeneous distribution of bacteria that could be *actinomyces*, with only isolated streptococci and veillonella forming small clusters. Treatment with the Amox/Metro combination results in a further increase in dead cells and a marked reduction in living cells; only few bacteria, probably *actinomyces* and streptococci are detectable, whereas veillonella-like bacteria appear more frequently in clustered arrangements. The Doxy-treated biofilm similarly contains only few streptococci but exhibits more pronounced clustering of *actinomyces* compared with Amox/Metro. In contrast, under Doxy/Metro treatment, streptococci-like bacteria appear to be largely preserved and no cluster formation is observed. The resulting biofilm is clearly more homogeneous than with the other AB treatments and resembles the growth control, albeit with a reduced cell density and a higher proportion of dead cells. The Metro-treated biofilm, by comparison, displays a high overall cell density, comprising both living and dead bacteria. All microbial species are present, with bacteria, that look like *actinomyces* appearing to constitute the largest proportion. Cells are distributed across the entire surface and frequently form clusters, which contain a relatively high number of living cells. Chlorhexidine (CHX) treatment led to a significant reduction of cells, only very few living and dead cells can be seen in the corresponding image.

**FIGURE 5:**
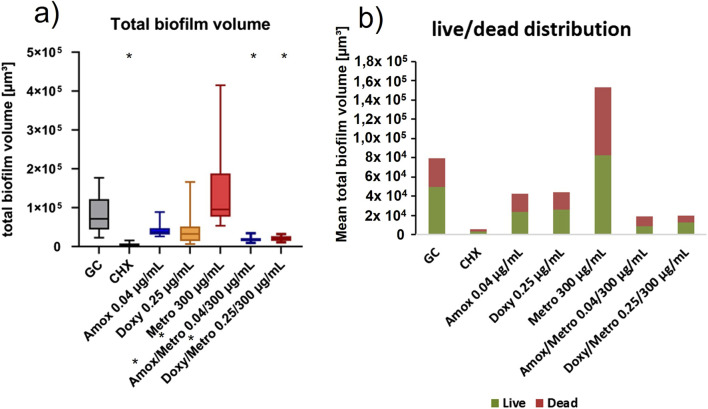
**(a)** total biofilm volume calculated from CLSM images after 24 h treatment with AB (combinations) and live/dead staining examined individually and given as Tukey boxplots. GC = growth control. * indicate significant difference from GC (top) respectively individual AB (bottom) (p ≤ 0.05). **(b)** mean values of live/dead distribution within the biofilm after 24 h treatment with AB (combinations) calculated from CLSM images after live/dead staining.

Biofilm volumes and live/dead distribution were further calculated for each image. Figure 5 provides an overview over the total biofilm volume (a) and the live/dead proportions (b) of each experimental group. Corresponding to the other results, the lowest total biofilm volume can be found with Amox/Metro and Doxy/Metro. Amox and Doxy alone still show reduced biofilm volumes compared to the growth control. However, Metro alone shows a significantly higher biofilm volume then the growth control. Although Amox and Amox/Metro show similar values in total biofilm like Doxy respectively Doxy/Metro, the combinations with Amox result in higher proportions of dead bacteria in the biofilm then those with Doxy.

### 3.4 Efficacy is independent from implant material

**FIGURE 6:**
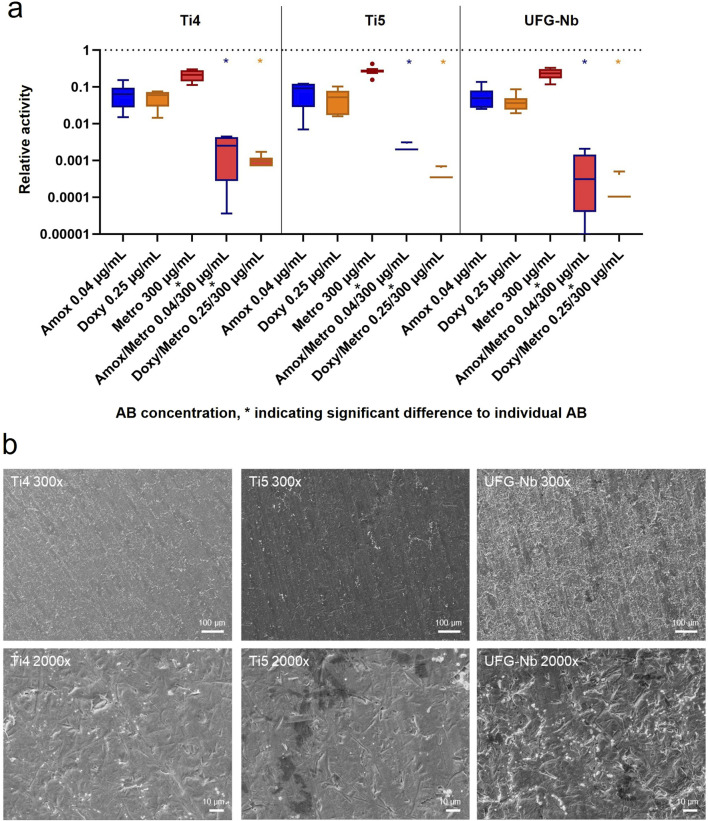
**(a)** Results of AB applied on MB formed on the surfaces of Ti4, Ti5 and UFG-Nb given as Tukey boxplots. Ti4 = grade 4 titanium, Ti5 = grade 5 titanium, UFG-Nb = ultrafine-grained niobium alloy, * = significant differences (p ≤ 0.05). **(b)** representative SEM visualization of the materials after bacterial culture at 300x and 2000x magnification. Longitudinal grooves from the sawing process to prepare specimens can be seen on all surfaces. Residual bacteria (ball-shaped strucures) are present on the surfaces. All materials show highly similar surface characteristics.

The most effective combinations–Amox/Metro 0.04/300 and Doxy/Metro 0.25/300 – along with their respective single-antibiotic components were further analyzed with MB formed on the surfaces of three potential implant materials (Ti4, Ti5 and UFG-Nb). The corresponding results are presented in Figure 6a. Comparable results were observed across all tested implant materials, with no statistically significant differences in AB efficacy between the different materials. All AB and AB combinations exhibited an effect on the MB. Importantly, the synergistic effects of the AB combinations were maintained across all tested implant materials. Consistent with the results of the first study parts, Metro alone showed the lowest efficacy, whereas Amox and Doxy demonstrated greater effects but did not achieve relative values below 0.01. Both tested AB combinations (Amox/Metro and Doxy/Metro) resulted in significantly lower values compared with the Metro alone. Across all materials, the median value for Doxy/Metro was slightly lower than that for Amox/Metro. When comparing the implant materials, the median values for both AB combinations were slightly lower for UFG-Nb than for Ti4 and Ti5. However, these differences were not statistically significant.

The exemplary SEM images are shown in Figure 6b. The micrographs of the implant materials reveal very similar surface structures across all three materials. As a result of the sawing process used for plate preparation, all surfaces display pronounced longitudinal grooves. Additionally, angular particles—most likely precipitated salts from the culture medium—and residual bacterial cells appearing as round structures are visible on the metallic surfaces.

### 3.5 Efficacy is dependent from biofilm maturation

**FIGURE 7:**
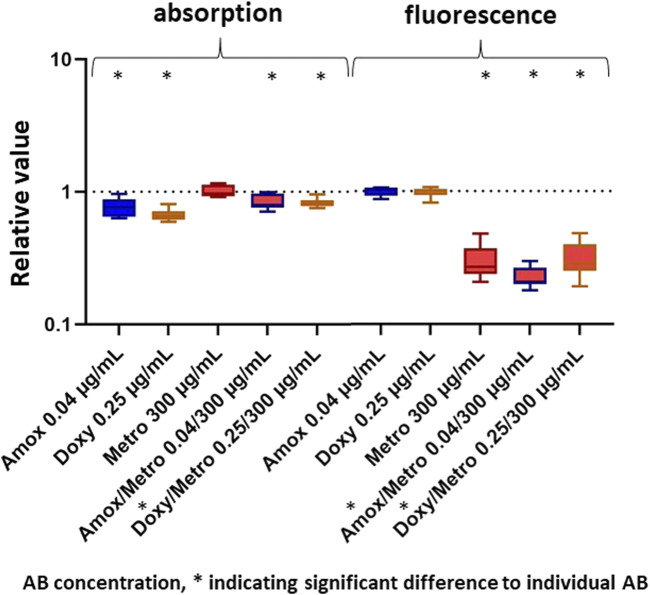
Results of AB applied on mature MB for absorption (left) and fluorescence (right) given as Tukey boxplots. * = significant differences (p ≤ 0.05).

The most promising combinations identified in the initial experiments were subsequently applied to a mature biofilm that had been cultivated for 24 h prior to AB treatment. The corresponding results are shown in Figure 7. All agents, with the exception of Metro, resulted in a significant reduction in biofilm growth (absorption) with the lowest relative mean value being 0.663 for Doxy. In contrast to the results obtained for immature biofilm, the combination of Doxy with Metro resulted in lower growth inhibition than the administration of Doxy alone (p = 0.04). However, the combination of Amox with Metro resulted in similar values like the respective single AB. With regard to bacterial activity (fluorescence), only Metro and the combined AB (Doxy/Metro and Amox/Metro) demonstrated significant efficacy. Amox and Doxy alone did not lead to reduced bacterial activity. Accordingly, the combined AB treatments resulted in significantly lower values than the respective single AB treatments (Amox or Doxy). Overall, the resulting values are substantially higher than those obtained for the immature biofilm in the first and second parts of the study.

## 4 Discussion

Nowadays, dental implants play a crucial role in the replacement of missing teeth; however, they are susceptible to biofilm-caused complications such as mucositis and peri-implantitis. Although the use of adjunctive antibiotics has been shown to improve clinical outcomes, the limited strength of current evidence and the growing concern regarding antimicrobial resistance have led international guidelines to discourage their routine use in the management of peri-implantitis (Barbato et al., 2023; Herrera et al., 2023; Liñares et al., 2023; Antonoglou et al., 2025). Consequently, determining the required antibiotic concentrations and identifying effective combinations for potential local peri-implantitis therapy remain of high relevance and were the focus of this *in vitro* study.

The AB used, Amox, Doxy, and Mino are broad-spectrum antibiotics with different antimicrobial spectra. As peri-implant infections are caused by a variety of bacterial species, the broad efficacy of the antibiotics employed is highly relevant and may be further enhanced by the additional administration of metronidazole. Our findings demonstrated that Amox and Doxy, whether applied individually or in combination with Metro, exhibit high efficacy against an MSBM. These observations align with current evidence, as Amox, alone or combined with Metro, remains the systemic regimen with the strongest support for improving peri-implantitis treatment outcomes (Barbato et al., 2023; Liñares et al., 2023; Luo et al., 2024; Antonoglou et al., 2025). Despite this, Amox and Metro have thus far been employed only infrequently in locally delivered peri-implantitis therapies. Instead, most investigations of locally applied antibiotics have focused on tetracycline derivatives, particularly Mino and Doxy (Toledano et al., 2021; Liñares et al., 2023). Local antibiotic delivery offers substantial advantages over systemic application, notably a reduced risk of systemic adverse effects. However, existing formulations often fail to achieve adequate retention, primarily due to biological clearance processes within peri-implant tissues (Nastri et al., 2019; Nakajima et al., 2021). Thus, our results suggest that both Doxy and Amox represent promising candidates for local application.

Amox is widely used in the management of odontogenic infections due to its high efficacy and low toxicity (Ahmadi et al., 2021). Its well-documented activity against both Gram-positive and Gram-negative bacterial species (LeCorn et al., 2007; Soares et al., 2015) is also reflected in our findings, that show reduced bacterial viability and activity in biofilms treated with Amox. However, owing to its extensive clinical application, penicillins such as Amox are increasingly threatened by the emergence of bacterial resistance. As a result, alternative broad-spectrum antibiotics, such as the tetracycline derivates Doxy and Mino have gained importance. Both AB exhibit comparable activity against a wide range of Gram-positive and Gram-negative germs and are frequently employed in peri-implantitis treatment, particularly in locally delivered formulations (Toledano et al., 2021; Liñares et al., 2023). Our findings likewise indicate strong efficacy of both compounds against oral MB and thus corroborate previously published data. In contrast, Metro demonstrates selective activity against anaerobic bacteria. This is why in peri-implantitis treatment it is usually administered in combination with other antibiotics, mostly Amox (López-Valverde et al., 2023). Consistent with these facts, Metro alone showed a low efficacy against the MSBM in our study, independent from the administered concentration. In contrast, the values for bacterial viability even increased, which is also visible in CLSM analysis, where biofilm treated with Metro shows by far the highest total biofilm volume. This could be explained by a stress reaction in the bacteria in presence of AB, but also by the low efficacy of Metro against aerobic germs such as *S. oralis* or *A*. *naeslundii* which are therefore able to proliferate even in the presence of Metro. CLSM analysis further substantiates this assumption, revealing a high level of *A. naeslundii* proliferation in biofilms exposed to Metro. A more detailed statement would, however, require a more detailed analysis of the individual components of the MSBM.

Correspondingly, all combinations of Amox, Doxy or Mino with Metro showed superior efficacy than Metro alone. Although every AB combination that achieved relevant antimicrobial effects (defined as relative values below 0.1) exhibited similar or higher efficacy than the corresponding antibiotic alone, none of these differences for Amox, Doxy, or Mino reached statistical significance. *Astasov-Frauenhoffer* et al. reported comparable findings in their experiments involving Metro and Amox applied to a three-species biofilm. Their analysis suggested that the observed synergistic effect may result from a modified mode of action, shifting from a primarily bacteriostatic to a more bactericidal behavior (Astasov-Frauenhoffer et al., 2014). Biofilm analysis using CLSM further supported the high efficacy of Amox and Doxy, particularly against the aerobic species *S. oralis*. In line with the results of the viability and activity assays, the combination of Amox and Metro resulted in a pronounced reduction in total biofilm volume and bacterial counts, most notably affecting those bacteria, that appeared like the aerobic organisms *S. oralis* and *A. naeslundii*. Amox and Doxy result in similar values for total biofilm volume, both alone and in combination with Metro. As expected, the biofilm volume observed for the combination treatments was significantly lower than that of the respective individual antibiotics. However, in terms of live/dead distribution, Amox shows a higher proportion of dead bacteria than Doxy, especially when comparing the combined AB. In line with this, compared with Amox, Doxy alone induced a less marked reduction of *A. naeslundii*, and its combination with Metro led to an overall decrease in living bacteria relative to the growth control, while only minor changes in biofilm composition were observed. Consistent with the literature, these results underline that Amox has a more bactericidal effect, whereas Doxy has a primarily bacteriostatic effect (Chopra and Roberts, 2001; Zeng and Lin, 2013). Further, these findings may indicate a superior efficacy of Amox against aerobic species, whereas Doxy appears to exert stronger effects on anaerobic organisms such as *V. dispar*. These observations concerning species distribution are based on a visual analysis of the biofilm. For more detailed analyses of AB efficacy on different bacterial species, other staining methods in CLSM or molecular methods, such as species-specific qPCR, would be necessary. As the investigated biofilm composition reflects primary implant colonization (Kommerein et al., 2017), further studies employing different biofilm models are required to substantiate this assumption. In contrast to the highly effective combinations of Metro with Amox and Doxy, the combinations of Metro with Mino did not demonstrate notable efficacy against the MSBM. Considering that the addition of Metro did not produce a significant enhancement in the other combinations either, this observation may be attributed to the comparatively less effective IC_P_ of Mino. In summary, the results indicate synergistic effects primarily when compared with Metro alone. Nonetheless, the data also reveal a tendency toward a supportive effect of Metro when combined with Amox and Doxy, which, although not statistically significant, is consistent with trends observed in several other *in vitro* studies and supported by the CLSM analysis (Pavicić et al., 1991; Astasov-Frauenhoffer et al., 2014; Belibasakis and Thurnheer, 2014; Soares et al., 2015; Rams and Faucher, 2025). This trend must be further investigated in future studies, for example, using specific methods such as fractional inhibitory concentration indices or mathematical models of pharmacological interactions.

With the exception of Amox, our data overall indicate a consistent trend toward bacterial responses to the respective AB occurring at lower concentrations in MS than in MB. Owing to the protective properties of biofilms, planktonic bacteria respond to AB exposure at lower concentrations than those within biofilms (Eick et al., 2004). Bacteria may exhibit increased metabolic activity as a stress response at low AB concentrations, whereas at higher concentrations, bacterial activity is reduced as a consequence of AB efficacy. Both effects can also be seen in our results on immature and mature biofilm. As peri-implant infections are biofilm-associated diseases, particular emphasis should therefore be placed on the MB-related results when considering clinical applicability as well as on the results achieved on mature biofilm. With respect to effective concentrations in the first study part, Amox exhibited its greatest activity against the MSBM at 0.25 μg/mL. These findings are consistent with previously reported minimal inhibitory concentration (MIC) for Amox, which range from approximately 8 μg/mL for *Veillonella* species (Alou et al., 2009) to values below 0.016 μg/mL for *P. gingivalis* (van Winkelhoff et al., 2005). To evaluate potential synergistic effects with Metro in combined applications, lower concentrations were used, defined as IC_P_ and IC_S_. For Doxy, maximal efficacy was observed at concentrations ≥1 μg/mL, while further increases above this level did not appear to enhance antimicrobial activity. Notably, significant reductions across all assessed parameters were already detectable at a Doxy concentration of 0.25 μg/mL. Published MIC values for Doxy span from 12.5 μg/mL for *P. gingivalis* (Eick et al., 2004) to 0.125 μg/mL for a four-species biofilm model (Park et al., 2014), placing our findings at the lower end of this reported range. This may be attributed to a comparatively high sensitivity of the bacterial species mainly composing our MSBM to Doxy, which has been documented for *A. naeslundii* (Feres et al., 1999) and various *Streptococcus* species (Rams et al., 2014), whereas *P. gingivalis*, according to *Eick* et al. (Eick et al., 2004), appears to exhibit lower susceptibility. Compared with AB concentrations typically achieved through systemic administration in the gingival crevice fluid, the effective concentrations identified in our study were considerably lower (Tenenbaum et al., 1997; Sakellari et al., 2000; Lavda et al., 2004; Pähkla et al., 2005). On the one hand, this finding could suggest that effective therapeutic outcomes may also be achieved with lower concentrations than those currently employed in clinical settings. On the other hand, the comparatively low bacterial diversity in our model may account for the increased sensitivity of the biofilm to antibiotic treatment. Accordingly, further studies incorporating biofilms with greater bacterial diversity are required to more precisely determine the clinically necessary concentrations. However, these findings reinforce the rationale that, for local applications, lower AB concentrations are sufficient and indeed should be employed compared to systemic administration. It can be assumed that the concentrations of Amox, Doxy, and Mino used in the present study exert a clinically acceptable cytotoxic effect, as these concentrations are substantially exceeded during systemic AB administration. In contrast, Metro was applied at a considerably higher concentration, which has been shown to induce cytotoxic effects on fibroblasts in a study by *Ferreira* et al. (Ferreira et al., 2010). To corroborate these assumptions and to allow for more precise conclusions with regard to the clinical situation, future investigations should employ experimental models of increased complexity. In particular, such models should incorporate human cells as well as a broader spectrum of bacterial species in order to more accurately approximate *in vivo* conditions.

This study also aimed to investigate the influence of implant materials on the effectiveness of the AB. To this end, the experiments were additionally performed in the presence of three potential implant materials: Ti4, Ti5, and the experimental UFG-Nb. Additionally, the materials were analyzed through SEM imaging, revealing similar surface characteristics and colonization patterns. Biological experiments revealed comparable values for the investigated AB (and AB combinations) both in the absence and presence of implant materials, with the highest efficacy observed for the combinations of Metro with Amox and Metro with Doxy. No significant differences were detected among the three materials, but in tendency the efficacy of both AB combinations was slightly higher on UFG-Nb then on Ti4 and Ti5. All three materials exhibit high biocompatibility and biological inertness, primarily due to their stable and thick oxide layers, which minimize the likelihood of chemical interactions with active pharmaceutical compounds or bacterial metabolites (Rubitschek et al., 2012; Pradhan et al., 2016; Hanawa, 2022; Safavi et al., 2022). The high compatibility of titanium is well established in the literature (Burgueño-Barris et al., 2021; Hanawa, 2022; Wang et al., 2025), and the findings of the present study further suggest a similarly or even higher degree of biological passivity for the experimental UFG-Nb. It should be noted, however, that variations in surface structure or the application of biologically active coatings may significantly influence both the prevention and treatment of peri-implantitis (Grischke et al., 2016; Burgueño-Barris et al., 2021; Wang et al., 2022b; Yu et al., 2024). Future studies should therefore focus on these aspects, particularly regarding the potential interactions of active surface coatings with antibiotics.

In order to better approximate the clinical situation, antibiotics were additionally applied on a mature biofilm. As expected, the results indicate that the active substances were significantly less effective against the mature biofilm than in the earlier parts of the study. Although all antibiotics except Metro resulted in a significant reduction in bacterial growth, this reduction remained clinically irrelevant with relative values far above 0.1. Interestingly, in this part of the study the antibiotic combinations did not exert a positive effect on bacterial growth. However, bacterial activity was reduced only by the combination treatments and by Metro alone. The effectiveness of Metro alone in reducing bacterial activity was also observed in the results of the first part of the experiment. These findings indicate that Metro, as well as the antibiotic concentrations Amox/Metro and Doxy/Metro, inhibit the activity of a proportion of the bacteria but do not substantially contribute to bacterial killing in this experimental setup. This observation suggests that the concentrations applied are insufficient to eliminate a mature biofilm by antibiotic therapy alone. Nevertheless, the results demonstrate that combination therapy with Amox or Doxy together with Metro appears more promising than the administration of single antibiotics. According to the literature, concentrations up to 100-fold higher than those required for planktonic bacteria may be necessary to achieve effective activity against mature biofilms (Eick et al., 2004; Park et al., 2014), which would be associated with a substantially increased risk of cytotoxic and systemic side effects (Ferreira et al., 2010). Accordingly, the results emphasize the role of antibiotics in clinical practice as an adjunctive measure accompanying the essential mechanical elimination of the biofilm.

The limitations of this *in vitro* study primarily relate to the considerably reduced number of four bacterial species included in the model compared with the clinical situation (Joshi et al., 2025b). Although this simplification limits the direct clinical relevance of the findings, it enables a greater reproducibility and high number of experimental repetitions and is therefore well suited for this type of medium-throughput investigation, in which multiple experiments were required to systematically identify effective antibiotic compositions and concentrations. Furthermore, the biofilm model used represents an early stage of microbial colonization according to its species composition (Kommerein et al., 2017), thereby limiting its ability to reflect the microbial complexity of peri-implantitis. Consequently, conclusions derived from these results regarding the clinical situation—particularly in advanced cases of peri-implantitis—should be drawn with caution. In order to provide more detailed information on the efficacy of the applied AB (-combinations) on biofilms with different bacterial compositions, modification of the study design would be necessary. To validate our findings of this *in vitro* study, future research must be, preferably using more complex *in vitro* or *in vivo* models and, ultimately, clinical studies.

## 5 Conclusion and outlook

The purpose of this study was to identify effective AB combinations and concentrations against a peri-implantitis MSBM *in vitro* and to assess the influence of implant materials on their efficacy. To this end, Amoxicillin, Doxycycline, Minocycline and Metronidazole were tested both as single agents and in combination with metro against a four-species MSBM in three maturity stages, alone and in presence of three potential implant materials. Our findings confirmed the efficacy of Amox, Doxy and Mino but not of Metro alone, against the MSBM. Combinations exhibited synergistic effects primarily when compared to Metro’s efficacy. Although without statistical significance in the current experimental setup, our data also indicated a tendency of Metro to enhance the efficacy of both Amox and Doxy in combination. The efficacy of the AB was independent of the implant material which confirms the biological passivity of both titanium and the experimental UFG-Nb alloy. Overall, our results confirm the efficacy of locally applied Amox and Doxy in combination with Metro for the treatment of peri-implantitis. Future studies are required to validate these findings and to provide recommendations for clinical application.

## Data Availability

The original contributions presented in the study are included in the article/supplementary material, further inquiries can be directed to the corresponding author.
